# Trained Immunity in Neutrophils and Mononuclear Phagocytes: Mechanisms and Pathophysiological Functions

**DOI:** 10.3390/cells15090752

**Published:** 2026-04-23

**Authors:** Wanying Li, Jialei Wei, Liyuan Li, Wei Sun

**Affiliations:** 1 College of Basic Medical Sciences, Key Laboratory of Pathobiology, Ministry of Education, Medical Basic Research Innovation Center of Airway Disease in North China, Jilin University, Changchun 130021, China; liwy2722@mails.jlu.edu.cn (W.L.); weijl2722@mails.jlu.edu.cn (J.W.); lyli22@mails.jlu.edu.cn (L.L.); 2 Department of Radiological Medicine, School of Public Health, Jilin University, Changchun 130021, China

**Keywords:** trained immunity, neutrophils, monocytes/macrophages, tumor immunology, infectious diseases, hypoxia, ischemia

## Abstract

**Highlights:**

**What are the main findings?**

- Trained immunity is driven by the synergy of epigenetic remodeling and metabolic reprogramming in innate immune cells.
- It is initiated in bone marrow progenitors, allowing short-lived neutrophils to carry lasting functional memory into circulation.

**What are the implications of the main findings?**

- Inducing trained immunity (e.g., via BCG or β-glucan) provides a novel strategy for anti-tumor therapy and broad-spectrum infection protection.
- Precise control is required to prevent maladaptive training that could worsen chronic inflammation or cause immune paralysis.

**Abstract:**

Trained immunity refers to the enduring functional reprogramming of innate immune cells after particular stimuli, driven by epigenetic and metabolic alterations that augment non-specific responses upon subsequent exposure. Neutrophils and monocytes/macrophages, as essential innate effectors, are crucial for the induction and control of trained immunity, which is the primary emphasis of this review. Neutrophils, the predominant circulating leukocytes, were historically considered incapable of memory owing to their brief lifespan. Emerging evidence indicates that trained immunity functions at the bone marrow progenitor level, influencing granulopoiesis to produce neutrophils with lasting functional modifications. This research offers new insights into neutrophil functions in infection, cancer, and inflammation. Monocytes and macrophages, characterized by phenotypic plasticity and tissue residence, function as conventional models of trained immunity. They experience direct peripheral reprogramming or emerge as primed descendants of trained bone marrow precursors, performing pro-inflammatory or reparative roles in malignancies, infections, and ischemia lesions. This study comprehensively outlines the regulatory mechanisms of trained immunity in these cells, clarifies their functions in various clinical situations, and examines therapeutic applications. Comprehending these pathways is crucial for elucidating the cellular foundation of innate immunological memory, uncovering its multiple functions in disease, and guiding innovative therapeutics aimed at granulopoiesis and monocyte-macrophage polarization.

## 1. Overview of Trained Immunity

The traditional immunological paradigm posits a clear distinction between the innate and adaptive immune systems, differentiated by their cellular constituents, activation timelines, response specificity, and ability to generate immunological memory [1]. Adaptive immunity is characterized by its antigen-specific memory, initiated by antigen-presenting cells (APCs) that process and present antigens to T and B lymphocytes. While antigens are defined by their capacity to bind to specific receptors, the initiation of a productive immune response requires additional costimulatory signals, typically triggered by the recognition of danger signals or pathogen-associated molecular patterns (PAMPs). This recognition subsequently triggers clonal expansion and differentiation, culminating in pathogen elimination and the establishment of a durable, antigen-specific memory reservoir [2]. In contrast, innate immunity has historically been viewed as a rapid, non-specific first line of defense that lacks long-term immunological memory.

While this schematic division has long dominated the field, the concept of trained immunity provides a unifying framework that integrates long-standing observations of innate memory with our broader understanding of host defense. For decades, phenomena such as the heterologous protection afforded by certain vaccines and the heightened resistance induced by microbial components remained poorly understood or were dismissed as immunological curiosities [3]. While these diverse observations were rooted in early concepts of non-specific resistance, they have only recently been consolidated into a solidary biological system termed “trained immunity”. It refers to the process wherein innate immune cells, encompassing macrophages and neutrophils, undergo enduring functional reprogramming following an initial stimulus, such as infection, vaccination, or exposure to specific microbial components. This reprogramming results in an augmented, non-specific immune response to subsequent encounters. This heightened state is not antigen-specific but is instead regulated by substantial epigenetic and metabolic alterations. Specifically, trained immunity is defined by the persistent accumulation of permissive histone modifications, such as H3K27ac and H3K4me3, at the promoters and enhancers of genes encoding inflammatory mediators. This open chromatin structure promotes increased transcriptional accessibility and is supported by a metabolic transition towards increased glycolysis and oxidative phosphorylation [4,5].

**Figure 1:**
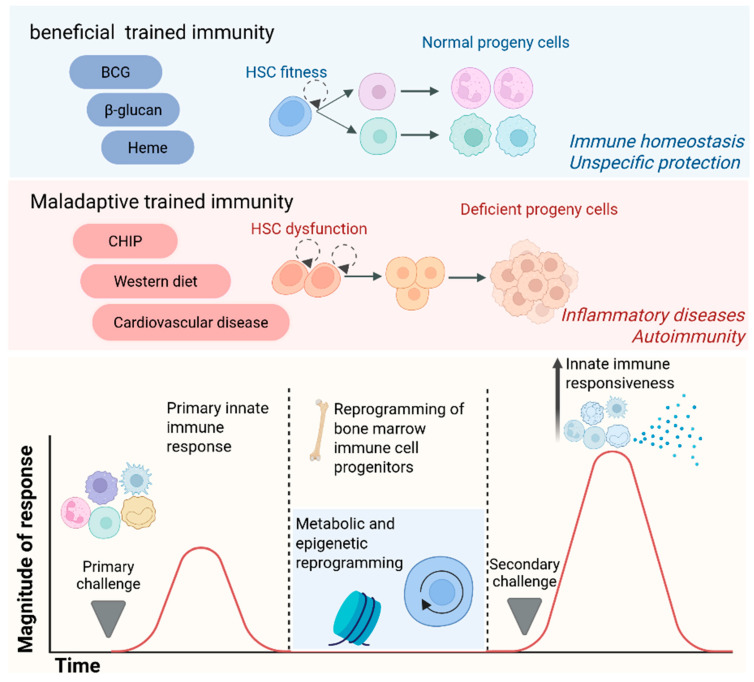
Schematic depiction of advantageous vs. detrimental trained immunity and its fundamental regulation mechanisms. (**Upper panel**): Beneficial trained immunity is elicited by classical stimuli, including Bacille Calmette-Guérin (BCG), β-glucan, and heme, which improve hematopoietic stem cell (HSC) fitness and sustain healthy differentiation and proliferation of hematopoietic progenitor cells. This process maintains immunological homeostasis and provides extensive, non-specific protection against various external threats. (**Middle panel**): Conversely, maladaptive trained immunity develops in reaction to pathogenic stimuli, such as clonal hematopoiesis of indeterminate potential (CHIP), a Western diet, and cardiovascular disease. These variables induce HSC malfunction, resulting in abnormal differentiation and the generation of functionally compromised progeny cells. This aberrant reprogramming disturbs immunological homeostasis and facilitates the onset of inflammatory and autoimmune disorders. (**Lower panel**): The cellular-level representation of the temporal dynamics of learned immunity is illustrated. Subsequent to an initial innate immune assault, a main response is activated. Thereafter, bone marrow immune progenitor cells experience prolonged metabolic and epigenetic remodeling, thereby developing enduring immunological memory. Upon subsequent challenge, these reprogrammed progeny cells demonstrate enhanced innate immune responsiveness, leading to a significantly intensified secondary response—a characteristic memory-like attribute of trained immunity. Adapted with permission from [6], copyright 2022 Elsevier Inc.

The induction of trained immunity embodies a dual nature with substantial implications for human health. This process confers a certain degree of heterologous protection against a wide array of infections, thereby augmenting the host’s baseline defensive capabilities. However, in characterizing trained immunity, a rigorous distinction must be maintained between its broad, relatively short-lived protection and the long-term, sterilizing immunity characteristic of the adaptive system. While trained immunity can enhance host survival during heterologous challenges, it does not achieve the level of precision required to neutralize highly virulent or specific pathogens, which necessitates dedicated vaccine-induced adaptive responses [6]. Conversely, the aberrant activation of trained immunity can instigate persistent, low-level inflammation, potentially fostering the emergence of diverse disorders such as atherosclerosis and autoinflammatory syndromes. Investigating these mechanisms reveals novel therapeutic targets for a variety of diseases (Figure 1).

## 2. Trained Immunity in Neutrophils, Monocytes and Macrophages

### 2.1. Trained Immunity in Neutrophils

Neutrophils represent the most abundant leukocyte population within human blood, comprising 50–70% of the total peripheral blood white cell count [7]. These cells, serving as the principal effector cells of the innate immune system, are crucial for executing fundamental antimicrobial defense strategies. The processes of their generation, maturation, and subsequent migration from the bone marrow to the peripheral circulation are highly regulated, collectively establishing the initial barrier of immune defense against pathogenic invaders. Neutrophils derive from hematopoietic stem cells (HSCs) in the bone marrow and progress through a series of differentiation stages, including multipotent progenitors (MPPs), common myeloid progenitors (CMPs), and granulocyte-monocyte progenitors (GMPs), culminating in the formation of neutrophil precursor cells [8]. These precursors advance through a sequence of morphologically and functionally diverse maturation stages before differentiating into adult segmented neutrophils, which can be mobilized and discharged into the peripheral circulation in response to infection or tissue injury. Mature neutrophils perform their immune effector tasks by many processes, including phagocytosis, generation of reactive oxygen species (ROS), formation of neutrophil extracellular traps (NETs), and secretion of pro-inflammatory cytokines, chemokines, and cytotoxic granules [9]. The processes of differentiation, maturation, and mobilization are highly coordinated biological activities regulated by complex signaling networks, cellular interactions, and epigenetic mechanisms inside the bone marrow microenvironment. Neutrophils have traditionally been regarded as ephemeral cells with tissue-specific differences in longevity. Studies on adoptive transfer and ex vivo survival indicate that neutrophils have circulatory half-lives of 8 to 12 h, with tissue persistence lasting 1 to 2 days [10,11].In vivo tracing methodologies indicate that human neutrophils may persist for as long as 5.4 days [12].

Increasing evidence suggests that neutrophils show significant differences in their structure and function depending on the changing conditions of their environment. This is especially clear in diseases like chronic inflammation, cancer, and sepsis. Neutrophil diversity is influenced by two main factors: internal differences that arise in the bone marrow and the different types of neutrophils that circulate, and external factors in the local or systemic environment that affect how neutrophils look and work. These multiple forces orchestrate precisely synchronized transcriptional and epigenomic processes, eventually determining the varied functional repertoires of neutrophils in both homeostasis and illness [8]. Induction of trained immunity has demonstrated that murine and human hematopoietic stem cells (HSCs) develop a lasting inclination towards granulopoiesis, resulting in enduring functional reprogramming of neutrophils [13]. Clarifying the processes and regulatory mechanisms governing neutrophil functional reprogramming within the framework of trained immunity will establish a vital theoretical basis for formulating therapeutic and preventive strategies aimed at modulating neutrophil effector functions in trained immunity.

Trained immunity causes a prolonged phenotypic alteration in circulating unstimulated neutrophils, evidenced by increased expression of activation markers and significantly decreased expression of immunosuppressive markers. The increase in activation markers, particularly CD11b and CD66b, indicates the priming of essential neutrophil effector functions—namely migration, degranulation, and respiratory burst initiation [14]—all of which are vital for pathogen elimination. In contrast, the substantial downregulation of the immunosuppressive marker PD-L1 is significant, as neutrophils exhibiting an immunosuppressive phenotype marked by elevated PD-L1 expression in the solid tumor microenvironment have been strongly linked to disease progression and diminished patient survival [15]. The findings indicate that altering neutrophil morphologies by the establishment of trained immunity could serve as a promising therapeutic approach in malignancy [16].

**Figure 2:**
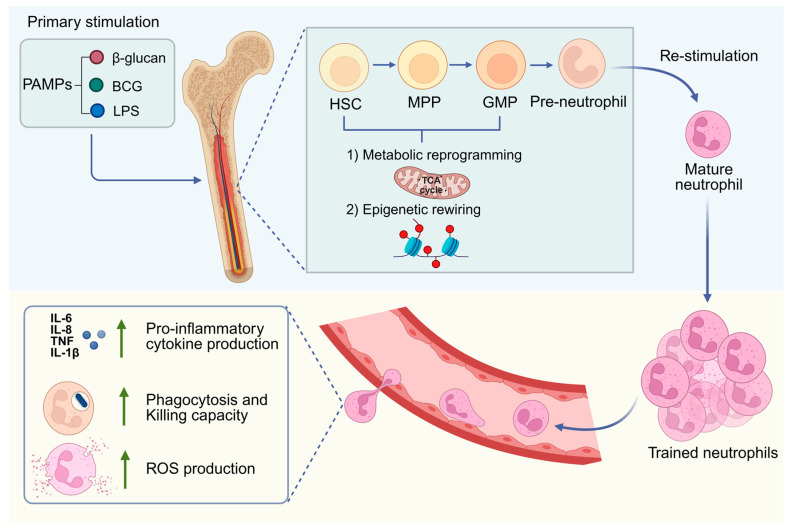
Induction Mechanisms and Functional Implications of Trained Immunity in Neutrophils. Upon initial stimulation with pathogen-associated molecular patterns (PAMPs), such as β-glucan, BCG, or LPS, hematopoietic stem cells (HSCs) and their progeny (multipotent progenitors [MPPs], granulocyte-monocyte progenitors [GMPs], and pre-neutrophils) in the bone marrow undergo two fundamental processes: (1) metabolic reprogramming, indicated by a transition from oxidative phosphorylation to aerobic glycolysis, and (2) epigenetic reconfiguration, signified by the accumulation of activating histone modifications (e.g., H3K4me3, H3K27ac) at pro-inflammatory gene loci. These molecular alterations create a durable, functionally reprogrammed condition in neutrophils. Mature neutrophils, upon secondary stimulation, demonstrate enhanced effector functions, characterized by increased production of pro-inflammatory cytokines (IL-6, TNF-α, IL-8, IL-1β), improved phagocytic and microbial killing abilities, and heightened generation of reactive oxygen species (ROS), collectively facilitating an intensified non-specific innate immune response. Figure created using Biorender.com.

Research indicates that trained immunity augments neutrophil responsiveness to subsequent infections. This phenomenon is mediated by epigenetic modifications and metabolic shifts, which in turn promote heightened production of reactive oxygen species (ROS), cytokine release, phagocytosis, and degranulation. Epigenetic mechanisms fundamentally govern the enduring memory characteristics of trained immunity. Initial stimulation, regardless of whether it involves β-glucan, Bacillus Calmette-Guérin (BCG), or lipopolysaccharide (LPS), triggers the activation of pattern recognition receptors (PRRs) [17]. This activation subsequently stimulates the production of histone-modifying enzymes. These enzymes then facilitate chromatin remodeling, which in turn affects the accessibility or condensation of particular gene regions [18]. The promoter regions of pro-inflammatory genes, including IL-6 and TNF-α, demonstrate an enrichment of activating histone modifications, such as H3K4me3 (trimethylation of histone H3 at lysine 4) and H3K27ac (acetylation of histone H3 at lysine 27). H3K4me3 has an intricate connection to active gene transcription, while H3K27ac is a hallmark of enhancer activity [3]. Considering the limited lifespan of circulating neutrophils and the established fact that the bone marrow reserve of granulocytes significantly surpasses their circulating numbers [19,20], it has been postulated that the functional and epigenetic changes observed in neutrophils after trained immunity may be facilitated by the epigenetic and functional reconfiguration of neutrophil progenitor cells. Subsequent investigations have demonstrated that, in steady-state conditions, the epigenetic alterations in neutrophils from trained individuals are negligible in comparison to those from untrained peers. These data indicate that primary stimulation promotes epigenetic alterations in neutrophil progenitors within the bone marrow. Upon subsequent challenge, these activated progenitors experience expedited maturation and are swiftly discharged into the bloodstream, facilitating a more effective response to microbial stimuli (Figure 2).

A further hallmark of trained immunity involves substantial modifications to cellular metabolic processes, specifically a shift from oxidative phosphorylation to aerobic glycolysis, a phenomenon recognized as the Warburg effect [21]. The mTOR-HIF-1α signaling pathway primarily controls this metabolic change. mTOR, or the mammalian target of rapamycin, is a key regulator of cell growth and metabolism. In contrast, HIF-1α, which stands for hypoxia-inducible factor-1α, helps cells adapt to low oxygen levels and changes in metabolism. The mTOR-HIF-1α pathway’s activation promotes glucose uptake and lactate production, thereby furnishing rapid energy and metabolic substrates that support heightened immune responses. Elevated lactate concentrations have been significantly associated with improved neutrophil functionality. Furthermore, compounds synthesized within the tricarboxylic acid (TCA) cycle, including succinate and itaconate, accumulate intracellularly. These metabolites, rather than being mere byproducts of energy metabolism, actively participate in epigenetic modifications. Succinate and itaconate inhibit histone demethylases, including KDM5, therefore preserving histone methylation at pro-inflammatory gene loci [22]. Recent research has shown that trained immunity enhances the expression of a new category of long non-coding RNAs (lncRNAs) in neutrophils, referred to as immunological priming lncRNAs (IPLs). In trained neutrophils, the presence of IL-1β increases H3K4me3 at the promoter regions of specific target genes, which in turn enhances gene transcription and antibacterial functions [23]. These findings clarify how trained immunity leads to specific epigenetic changes at different gene promoters.

In summary, using trained immunity to enhance neutrophil effector activities shows potential for mitigating the adverse effects of neutropenia. Alternatively, regulating neutrophil function through the inhibition of trained immune systems may present innovative treatment options for conditions marked by neutrophil-driven inflammation.

### 2.2. Trained Immunity in Monocytes and Macrophages

Monocytes are generally understood as a developmental stage, situated between bone marrow progenitor cells and tissue macrophages [24]. These cells, which originate in the bone marrow, then enter the bloodstream, where they circulate and migrate, thus contributing to tissue homeostasis through repair processes or by initiating inflammatory responses during infections [25,26]. In contrast, macrophages are terminally differentiated cells that reside in various tissues and organs throughout the body. Certain groups of tissue-resident macrophages originate from yolk sac-derived embryonic progenitors [27] and are maintained through local self-renewal; conversely, other populations, including those in the dermis and gut, are constantly replenished by adult monocyte-derived macrophages [28,29,30]. Moreover, in reaction to injury or infection, inflammatory monocytes from the adult bloodstream migrate to the affected tissues and differentiate into monocyte-derived macrophages, which demonstrate significant plasticity influenced by local microenvironmental signals [31,32]. Macrophages are capable of differentiating into specific phenotypes in response to microenvironmental cues, encompassing pro-inflammatory M1 macrophages and anti-inflammatory, pro-repair M2 macrophages [33]. This adaptability is essential for maintaining tissue homeostasis, combating infections, and regulating the progression of disease [34].

A variety of pathogen-associated molecular patterns (PAMPs) and damage-associated molecular patterns (DAMPs) can elicit trained immunity in monocytes and macrophages. Among these, β-glucan sourced from fungal cell walls, functioning via Dectin-1 receptor-mediated signaling pathways, constitutes a well-established model [35]. Moreover, the extensive protection provided by Bacillus Calmette-Guérin (BCG) immunization against diverse diseases is mostly due to the activation of trained immunity in monocytes and macrophages [6]. In recent years, some viral infections or vaccines—such as inactivated SARS-CoV-2 vaccinations and non-replicating viral vector vaccines—have demonstrated the ability to elicit a regulatory trained phenotype in human monocytes [36]. Endogenous risk cues can similarly elicit learned immunity. Oxidized low-density lipoprotein (oxLDL) activates monocytes and macrophages, promoting a pro-inflammatory phenotype and contributing to atherosclerosis pathogenesis [37], indicating that trained immunity may have a harmful function in chronic non-communicable diseases.

The concentration of histone modifications at gene promoters is a pivotal event in the regulation of trained immunity, particularly marked by histone H3 lysine 4 trimethylation (H3K4me3) and histone H3 lysine 27 acetylation (H3K27ac) [38]. These alterations preserve an open chromatin structure at inflammation-associated genes (e.g., TNF, IL-6), hence enhancing their accessibility for transcription [18]. As a result, trained cells demonstrate a more expedited and vigorous transcriptional activation of these genes upon subsequent stimulation. As a result, this process leads to more cytokine release, improved phagocytic and bactericidal functions, and a change in the functional polarization setpoint. Metabolically, monocytes and macrophages undergo significant changes at the cellular level when exposed to training signals through pattern recognition receptors. This is mainly characterized by a shift from oxidative phosphorylation to aerobic glycolysis. Although aerobic glycolysis is less efficient in terms of ATP production per glucose molecule, it allows for quick ATP production and provides biosynthetic precursors like acetyl-CoA [39]. This metabolic shift is critical for regulating epigenetic reprogramming, mainly by allowing metabolic intermediates to directly affect the activity of histone-modifying enzymes. The mTOR signaling pathway governs key aspects of the metabolic reprogramming associated with trained immunity, resulting in elevated nuclear acetyl-CoA levels that promote histone acetylation [40]. Furthermore, trained immunity is closely linked to alterations in mitochondrial function; for example, fumarate-induced trained immunity modulates mitochondrial activity, thereby influencing the cell’s capacity to withstand infection [41].

The establishment of trained immunity in monocytes and macrophages produces paradoxical biological consequences, augmenting host defensive mechanisms while potentially contributing to the pathogenesis of chronic inflammatory disorders. The most significant protective effect is heterologous protection; for example, Bacillus Calmette-Guérin (BCG) vaccination decreases overall mortality from neonatal sepsis and respiratory infections, an effect partially due to the heightened responsiveness of trained monocytes and macrophages to non-specific pathogens [6]. β-Glucan can reprogram macrophages in the tumor microenvironment, transforming them from a pro-tumor M2-like phenotype to an anti-tumor M1-like phenotype, hence enhancing immune surveillance [42]. Conversely, when improperly activated or maintained, trained immunity may exacerbate disease processes. Atherosclerosis involves chronic stimuli within the vascular wall, including oxidized low-density lipoprotein (oxLDL) and cholesterol crystals, which perpetually activate circulating monocytes and plaque-resident macrophages, keeping them in a prolonged pro-inflammatory primed state that intensifies local inflammation and fosters plaque instability [37,43]. Similar pathways function in other chronic metabolic inflammatory disorders, such as obesity, type 2 diabetes, and gout. Moreover, trained immunity could potentially worsen autoimmune diseases. This happens by lowering the activation threshold of the immune system, which makes people more likely to have stronger immune reactions to their own antigens [44].

Despite these well-documented biological consequences, the clinical manifestation of trained immunity specifically within the human monocyte and macrophage lineage remains characterized by substantial heterogeneity. Although mechanistic studies in cellular and animal models provide robust support for its therapeutic potential, results from large-scale human trials, including those investigating the heterologous effects of BCG, have occasionally been inconsistent. To clarify these complexities and address the concerns raised by clinical evidence, we have summarized the key findings, limitations, and conflicting results emerging from human studies of trained monocytes and macrophages in Table 1.

Specifically, several major challenges in clinical translation persist. First, the trained monocyte phenotype may vary significantly between in vivo systemic exposure and subsequent ex vivo differentiation, highlighting a notable context-dependency. Second, assessing central trained immunity in humans often relies on peripheral blood monocytes as a surrogate because repeated bone marrow sampling is ethically restricted. Third, significant inter-individual variability exists as factors such as age, sex, and prior microbial exposure modulate the magnitude of training, which complicates the standardization of these findings across diverse populations.

### 2.3. The Origin of Innate Memory: Central Trained Immunity vs. Peripheral Reprogramming

The debate regarding whether trained immunity is an intrinsic property of mature effector cells or a consequence of progenitor reprogramming is central to our understanding of innate immunological memory. Given the rapid turnover of myeloid cells, recent studies increasingly support a model where memory is primarily “stored” in the bone marrow (BM) and then transmitted to progeny, though local tissue-specific training also plays a distinct role [51].

As neutrophils typically survive for only a few days in circulation, the long-term effects of trained immunity are largely attributed to trained granulopoiesis—the functional and metabolic expansion of myeloid progenitors in the BM [52]. Recent evidence demonstrates that systemic priming (e.g., by β-glucan or TLR ligands) induces a “myeloid-biased” state in hematopoietic stem and progenitor cells (HSPCs) through the activation of the IL-1β and GM-CSF signaling axes [53]. Rather than neutrophils themselves remembering a stimulus, it is the Granulocyte-Monocyte Progenitors (GMPs) that undergo stable epigenetic remodeling. This education in the BM niche ensures that newly produced neutrophils possess a pre-activated transcriptional profile, characterized by enhanced ROS production and cytokine release, even weeks after the initial stimulus has been cleared [54]. Thus, referring to this phenomenon as “trained granulopoiesis” may more accurately reflect the biological level at which the memory is stored compared to the term “trained neutrophils”.

For monocytes and macrophages, memory storage is even more complex, involving both central and peripheral compartments. Central trained immunity involves the epigenetic reprogramming of HSCs, ensuring that circulating monocytes inherit a primed status before they even enter the blood [55]. Recent high-resolution single-cell studies have confirmed that BCG vaccination or inflammatory stimuli can alter the chromatin accessibility of HSCs, a change that persists and is transmitted during differentiation [56]. However, this does not diminish the importance of peripheral trained immunity. Mature tissue-resident macrophages (such as alveolar macrophages or Kupffer cells) can be trained directly by local environmental cues without the involvement of BM progenitors [57]. For instance, local respiratory viral infections can reprogram lung macrophages into a long-lived trained state that provides heterologous protection against bacterial secondary infections, a process that relies on local IFN-γ signaling rather than systemic HSPC alteration [58].

The clinical outcome of progenitor-driven memory depends heavily on the specific context and the duration of the initial stimulus. Under conditions of host defense or malignancy, trained granulopoiesis acts as a protective mechanism. For instance, it enhances the anti-tumor activity of neutrophils and monocytes by ensuring a steady supply of effector cells with heightened cytotoxic potential. Conversely, this central imprinting can become profoundly pathogenic within the environment of chronic inflammatory states. Recent research highlights that maladaptive trained immunity in HSPCs contributes to the persistence of cardiovascular diseases and metabolic disorders. This occurs because the bone marrow generates a continuous and long-lasting supply of hyper-inflammatory myeloid cells, which exacerbate tissue damage [59]. Therefore, understanding these distinct storage levels, particularly the contrast between central progenitor imprinting and local tissue adaptation, is crucial for developing precision therapies.

## 3. The Roles of Trained Neutrophils and Macrophages in Antitumor Immunity

### 3.1. Trained Neutrophils: Antitumor Potential and Pro-Tumor Risks

Myeloid lineage cells constitute a substantial component of the immune cell infiltration observed within tumors [60]. The tumor microenvironment exerts a functional reprogramming effect on tumor-infiltrating immune cells, promoting myeloid cell proliferation and often steering their polarization towards specific tumor-associated phenotypes [61]. Neutrophils, as crucial immune cells of the myeloid lineage, exhibit dynamic and complex phenotypic and functional modifications, demonstrating functional heterogeneity that is dependent on the particular cancer type they encounter [62,63]. Tumor-associated neutrophils (TANs), which are neutrophils that migrate into solid tumors, can differentiate into two main types: one with cytotoxic and anti-tumor properties (TAN1), and another associated with tumor growth (TAN2) [64]. Therefore, immunotherapy strategies that aim to reduce the tumor-promoting effects of innate immune cells, or that encourage their change into an anti-cancer form, represent a promising area of cancer treatment. These strategies could potentially improve the effectiveness of current immunotherapies that target adaptive immunity, thus increasing their ability to fight cancer.

Research has shown that trained immunity agonists exhibit anticancer efficacy. Bacillus Calmette-Guérin (BCG) has been therapeutically utilized in the treatment of bladder cancer [65], whereas β-glucan has been significantly linked to the effectiveness of cancer immunotherapy [66]. BCG immunotherapy for bladder cancer constitutes one of the most promising immunotherapeutic approaches for this malignancy. Research indicates that neutrophils are essential in BCG-mediated anticancer mechanisms; during BCG stimulation, neutrophils secrete tumor necrosis factor-related apoptosis-inducing ligand (TRAIL), a protein crucial for triggering tumor cell apoptosis. Furthermore, BCG-activated neutrophils secrete neutrophil extracellular traps (NETs), predominantly consisting of DNA, elastase, and histones [67]. BCG facilitates anticancer immune responses by prompting neutrophils to eliminate tumor cells by phagocytosis and degranulation.

**Figure 3:**
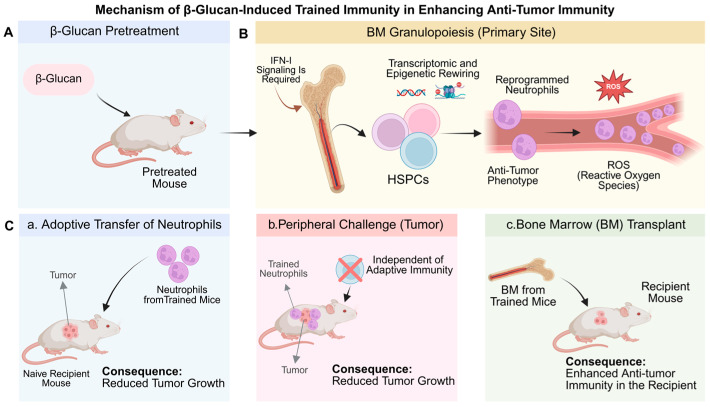
Innate immune training of granulopoiesis promotes anti-tumor activity. (**A**) β-Glucan pretreatment initiates trained innate immunity in mice, with type I interferon (IFN-I) signaling as an essential regulator. (**B**) Hematopoietic stem and progenitor cells (HSPCs) in the bone marrow (BM) undergo transcriptomic and epigenetic rewiring as a result of β-glucan and IFN-I signaling, producing reprogrammed neutrophils with an anti-tumor phenotype marked by elevated production of reactive oxygen species (ROS). (**C**) The causative anti-tumor impact is confirmed by three in vivo experiments: (a) adoptive transfer of neutrophils from trained mice suppresses tumor growth in naive recipient mice in a ROS-dependent manner; (b) peripheral tumor challenge shows that trained neutrophils inhibit tumor growth independently of adaptive immunity (T/B cells); (c) BM transplantation from trained mice confers sustained enhanced anti-tumor innate immunity to recipient mice, demonstrating the transmissibility of trained immune memory via hematopoietic stem cells. Figure created using Biorender.

Induction of trained immunity via β-glucan has been shown to enhance neutrophil-mediated anti-tumor surveillance through the reprogramming of granulopoiesis. A study has shown that β-glucan triggers transcriptomic and epigenetic rewiring of bone marrow granulocyte progenitors, leading to the generation of neutrophils with a sustained anti-tumor phenotype characterized by heightened ROS production and improved antigen presentation. Notably, the transferability of this protective trait through bone marrow transplantation confirms that the ‘memory’ of trained neutrophils is imprinted at the progenitor level rather than in short-lived mature cells. This mechanism of central training is further supported by human single-cell RNA sequencing data, which identifies a comparable enrichment of trained granulopoiesis signatures in patients, providing a robust clinical basis for targeting innate memory in cancer immunotherapy [52] (Figure 3).

In terms of pharmacological induction of trained immunity, a study developed MTP-PE-functionalized high-density lipoprotein nanoparticles (MTP-HDL), leveraging NOD2 agonism to systematically induce trained immunity. This approach significantly enhanced the efficacy of immune checkpoint inhibitors across various refractory murine tumor models, including melanoma, lung, breast, and pancreatic cancers. Mechanistically, MTP-HDL treatment triggered metabolic reprogramming—characterized by upregulated GLUT-1 expression and increased glycolytic dependency—alongside a shift toward a pro-inflammatory transcriptomic profile in neutrophils within the bone marrow, spleen, and tumor microenvironment. While currently in the preclinical stage, this study provides a crucial proof-of-concept for utilizing drug-induced neutrophil training to overcome tumor-mediated immunosuppression [68].

Tumor-derived signals can systematically reprogram neutrophils into a trained state with potent anti-tumor properties. Clinical data from breast cancer patients revealed that peripheral neutrophils undergo tumor-driven entrainment (TENs) mediated by CCL2, CCL5, and CXCL12. These cells exhibit sustained NADPH oxidase-dependent cytotoxicity and can inhibit lung metastasis upon adoptive transfer. This landmark study provides human evidence that the tumor environment can function as a training stimulus, imprinting neutrophils with a persistent anti-metastatic memory [69].

Current evidence underscores the pro-tumorigenic potential of trained neutrophils, which is driven by both systemic conditioning and localized microenvironmental reprogramming. Recent studies in humanized models and clinical cohorts have revealed that tumor-derived systemic factors, such as G-CSF and Flt3L, induce a form of central trained immunity by expanding myelocyte (MC) and metamyelocyte (MM) stage neutrophils in the bone marrow. These immature subsets, characterized by sustained immunosuppressive properties, subsequently infiltrate the tumor as a dominant subpopulation, thereby facilitating cancer progression through altered granulopoiesis [70]. Furthermore, within the tumor microenvironment (TME), neutrophils undergo further functional specialization. As demonstrated by Ng et al. in pancreatic cancer, neutrophils—irrespective of their initial maturation state—converge into a terminally differentiated dcTRAIL-R1^+^′T3′ subset. This transformation is marked by distinct epigenetic hallmarks, including altered chromatin accessibility and metabolic rewiring toward glycolysis, resulting in an extended lifespan and pro-angiogenic activity. Crucially, the clinical relevance of this trained T3 signature has been validated through human single-cell transcriptomics and TCGA pan-cancer analyses, where its prevalence strongly correlates with adverse patient prognosis [71]. Together, these findings illustrate that neutrophils undergo a multi-layered training process, from bone marrow precursors to terminally differentiated effectors, suggesting that targeting these specific reprogramming axes offers a promising therapeutic avenue to counteract tumor-mediated immune evasion.

In summary, the interaction between neutrophils elicited by trained immunity and malignancies is largely context-dependent. A comprehensive examination of the metabolic regulation and signaling pathways involved offers potential for leveraging the antitumor capabilities of neutrophils while mitigating their pro-tumor effects, thus establishing a theoretical basis for the advancement of more effective integrated cancer immunotherapeutic strategies.

### 3.2. Macrophage-Mediated Antitumor Immunity via Trained Immunity

Tumor-associated macrophages (TAMs) represent a predominant subset of immune cells in the tumor microenvironment. They can be classified into tissue-resident macrophages and circulating monocyte-derived interstitial macrophages based on their origin [72]. Some tissue-resident macrophages can directly convert into pro-tumor tumor-associated macrophages (TAMs) during the initial stages of carcinogenesis, influenced by the tumor microenvironment [73]. Circulating monocytes are the principal source of tumor-associated macrophages (TAMs); they are drawn to tumor sites by chemokines released by tumors, such as CCL2 and CSF-1, with substantial recruitment of bone marrow-derived monocytes occurring at these sites. Regulated by intricate signals within the tumor microenvironment, including IL-4, IL-10, IL-13, and TGF-β, these monocytes differentiate into macrophages and polarize into tumor-associated macrophages (TAMs), which perform functions such as immunosuppression, angiogenesis promotion, and facilitation of tumor invasion and metastasis [74]. TAMs exhibit significant phenotypic flexibility. Their phenotype generally matches that of M2 macrophages (alternatively activated), principally performing immunosuppressive actions and facilitating tissue repair and angiogenesis [75]. Under some settings or via targeted interventions, TAMs can be reprogrammed to display anticancer M1-like macrophage activities, including improved antigen presentation, secretion of pro-inflammatory cytokines, and direct cytotoxicity against tumor cells [76]. This phenotypic flexibility serves as a crucial theoretical basis for anticancer medicines aimed at targeting tumor-associated macrophages (TAMs).

Studies indicate that the creation of the pre-metastatic niche depends on substances released into the bloodstream by primary tumors. In particular, activating myeloid cells and related processes is crucial for creating a microenvironment that supports the survival and growth of metastatic tumors [77,78]. Therefore, enhancing immune surveillance by changing myeloid cells to an anticancer state could potentially limit or control the spread of tumors. In therapeutically pertinent lung metastasis models, trained immunity elicited by particle β-glucan (WGP) significantly inhibited tumor spread and extended host life. This effect is primarily contingent upon the specific expansion and phenotypic alteration of bone marrow-derived lung interstitial macrophages, which acquire a trained immune phenotype, thereby enhancing their phagocytic capacity and cytotoxic function against tumor cells upon exposure to tumor-derived factors. The mechanism at play involves WGP’s stimulation of the sphingolipid synthesis pathway, which leads to an increase in sphingosine-1-phosphate (S1P). This buildup then activates Drp-1, initiating mitochondrial fission, which in turn raises the production of mitochondrial reactive oxygen species (mtROS), thereby enhancing the antitumor effector functions of macrophages [79]. As a result, the induction of trained immunity via WGP treatment could offer a viable strategy for reducing secondary organ metastasis in the early stages of cancer.

In an orthotopic mouse model of pancreatic ductal adenocarcinoma (PDAC), peripheral trained immunity elicited by WGP significantly altered the tumor microenvironment, decreased tumor burden, and extended host life. The antitumor effect is ascribed to WGP-induced substantial infiltration of monocytes/macrophages into the pancreatic region through the CCR2/CCL2 axis, with the infiltrated myeloid cells displaying distinctive traits of trained immunity, notably increased phagocytic ability and cytotoxic activity against pancreatic tumor cells [80]. These preclinical findings are beginning to translate into clinical applications. A phase II clinical trial evaluated the efficacy of irreversible electroporation (IRE) combined with oral β-glucan in patients with locally advanced pancreatic ductal adenocarcinoma (PDAC). The results demonstrated a median disease-free interval of 18 months and a median overall survival of 32.5 months in the combination group. These survival benefits were accompanied by significant immunological shifts, specifically a decrease in naïve T cells and an enrichment of terminal effector T cells, which correlated strongly with improved clinical outcomes. This trial provides preliminary clinical evidence that β-glucan-induced trained immunity can be harnessed as a viable therapeutic strategy for pancreatic cancer [81].

Research utilizing mouse models has shown that acute respiratory viral infections, including influenza A virus (IAV), can elicit tissue-specific trained immunity in resident alveolar macrophages (AMs) in the lung, leading to markedly improved phagocytic ability against tumor cells and providing lasting antitumor immunity in the lung [82]. In the course of tumor progression, an immunosuppressive microenvironment is formed in the lung, serving as a crucial mechanism for tumor immune evasion; however, research indicates that trained tissue-resident alveolar macrophages can withstand the regulatory effects of the tumor-associated immunosuppressive microenvironment at both transcriptional and epigenetic levels, at least until the mid-stage of lung tumor development [83]. Moreover, AMs with characteristics akin to trained immunity have been detected in human non-small cell lung cancer tissues [84] and their existence is strongly associated with antitumor immune activity. Consequently, eliciting trained immunity in tissue-resident macrophages may provide a potential treatment approach with enduring anticancer effects.

Cancer immunotherapy has led to the creation of many cancer vaccines, which are designed to boost T cell responses against tumors [85,86]; however, the effectiveness of these vaccines is often limited by the immunosuppressive environment created by the tumor [87]. Recent research suggests that changing the tumor microenvironment through trained immunity could significantly improve how well cancer vaccines work. Scientists have developed a specifically designed vaccine (BG/OVA@EcN) that employs inactivated probiotic EcN as a carrier, which is co-loaded with tumor antigens and the trained immunity inducer β-glucan (BG). This vaccine is efficiently internalized by macrophages at the injection site, thereby inducing trained immunity within these cells, which in turn enhances dendritic cell antigen presentation and CD8^+^ T cell activation, thus initiating adaptive immunological responses. This vaccination simultaneously elevates the levels of trained monocytes/macrophages in peripheral circulation, which, upon infiltrating tumor tissues, develop into an M1-like macrophage phenotype, therefore modifying the immunosuppressive tumor microenvironment [88].

In the CT26, MC38, and immunosuppressive Colon-26 colorectal cancer models, the combination of β-glucan-induced trained immunity and the PeptiCRAd vaccination significantly suppressed tumor growth [89]. This effect is elucidated by a complex cellular cascade: activated macrophages continuously secrete CXCL9/CXCL10, which, via the CXCR3 receptor, recruits and activates natural killer (NK) cells; subsequently, NK cells release CCL5, thereby facilitating the accumulation and activation of conventional type 1 dendritic cells (cDC1) within tumor tissues, ultimately leading to a considerable expansion of tumor-specific CD8^+^ T cells. This method effectively combines the body’s natural and learned immune responses, leading to lasting anti-tumor effects. As a result, it establishes a new approach for creating personalized cancer vaccines.

In summary, trained immunity in macrophages and neutrophils plays a crucial regulatory role in tumor immunology. Macrophages play a key role in battling cancer by transforming tumor-associated macrophages, or TAMs. This transformation curtails both the growth and metastasis of tumors, while simultaneously enhancing the efficacy of immunotherapy treatments. Trained immunity in neutrophils enhances the development of an anticancer N1 neutrophil phenotype. These two cell types can produce synergistic effects via common inducers, intercellular communication, and co-reprogramming of bone marrow progenitors, collectively altering the tumor microenvironment and hence affecting tumor growth and therapeutic responses [90]. Subsequent research must clarify the precise regulatory mechanisms governing trained immunity in these two cell lineages and their interactions within the tumor microenvironment, thus establishing a theoretical basis for the advancement of more effective cancer immunotherapeutic strategies.

## 4. Trained Immunity in Neutrophils and Macrophages: Implications for Infectious Diseases

### 4.1. Regulation of Neutrophils by Trained Immunity in Infectious Diseases

Neutrophils, the predominant leukocytes in circulation, serve as the initial line of defense against microbial invasion [91]. Trained immunity modulates essential neutrophil effector functions—such as phagocytosis, degranulation, and the production of inflammatory cytokines—mainly via epigenetic reprogramming and metabolic remodeling. Evidence from human studies reveals that BCG vaccination induces significant epigenetic modifications in peripheral blood neutrophils across multiple immune-related pathways. Specifically, neutrophils from vaccinated individuals exhibit elevated H3K4me3 levels at the promoter regions of genes involved in the JAK-STAT signaling pathway, pro-inflammatory cytokine production, and glycolysis. These epigenetic hallmarks suggest that neutrophils are primed in a state of enhanced transcriptional readiness, enabling a more potent response upon subsequent challenges. Notably, under specific pathological conditions, metabolic reprogramming associated with trained immunity may exhibit dual effects [92]. In diabetes, neutrophils are inclined to generate neutrophil extracellular traps (NETs) by ACLY-mediated acetylation; while this mechanism may intensify local inflammation, proper regulation can markedly improve bacterial clearance efficacy [93]. Further studies indicate that trained immunity not only modulates the functional state of mature neutrophils but also mediates more enduring effects through the reprogramming of hematopoietic stem and progenitor cells (HSPCs). For instance, β-glucan can reprogram hematopoietic stem cells (HSCs) via type I interferon signaling, inducing the expansion of a specific regulatory neutrophil subset. These cells exhibit an immature phenotype (CD101low, Ly6Glow) and undergo a metabolic shift from conventional glycolysis toward mitochondrial oxidative phosphorylation, characterized by a significant increase in mitochondrial mass and high expression of IL-10. In influenza A virus infection models, this population substantially enhances host disease tolerance [94]. A study employed zebrafish models to illustrate that hematopoietic stem and progenitor cells (HSPCs) experience mitochondrial metabolic reprogramming, induced by the transcription factor C/EBPβ, after primary infection. This reprogramming subsequently equips the neutrophils produced with augmented reactive oxygen species (ROS) production capability and intracellular bactericidal efficacy during secondary infection [54].

Sepsis is a severe disorder defined by systemic inflammatory response syndrome (SIRS) and multiple organ dysfunction syndrome (MODS) [95], with disease progression and patient prognosis directly dependent on neutrophil functionality [96]. This acute infection can elicit trained immunity by immunological reprogramming at the hematopoietic level in the bone marrow, significantly affecting neutrophil differentiation and function [97]. Clinically, the “left shift” (an increased proportion of immature neutrophils) in complete blood counts during severe infections and the proliferation of immunosuppressive granulocyte subsets are strongly correlated with a heightened risk of hospital-acquired infections, indicating that severe infections may provoke maladaptive trained immunity, resulting in prolonged immune dysfunction [98,99]. Kwok et al. established that STAT3-CEBPB-mediated emergency granulopoiesis is a fundamental characteristic of the sepsis endotype SRS1. This signaling pathway functions as an immediate compensatory mechanism in response to infection; however, it could also promote maladaptive reprogramming of the innate immune system. This reprogramming makes hematopoietic stem and progenitor cells (HSPCs) more likely to generate dysfunctional myeloid cells, which in turn contribute to prolonged immune paralysis. Consequently, this elucidates the core mechanism underlying persistent immunological impairment observed in specific sepsis survivors, even after the eradication of pathogens [97].

Trained immunity can, prior to infection, pre-activate neutrophils, thereby improving their capacity to clear pathogens and regulate inflammation before sepsis onset; these effects are contingent upon the host’s initial immune condition and the local inflammatory environment [100]. Pretreatment with CG or β-glucan enhances the myelopoietic potential and immunological responsiveness of innate immune cells [51,101]. As an illustration, BCG protects neonatal mice from polymicrobial sepsis by inducing emergency granulopoiesis and activating G-CSF signaling; conversely, this protective effect was not observed in an LPS-induced endotoxin shock model [100]. In instances such as necrotizing enterocolitis, trained immunity may exacerbate disease progression by intensifying inflammatory responses. Therefore, the precise modulation of trained immunity in sepsis represents a crucial focus for future investigations, highlighting the need to enhance antimicrobial effectiveness while mitigating excessive inflammatory damage and effectively preventing the onset of immunological paralysis in the later stages of the disease.

Bacillus Calmette-Guérin (BCG), lipopolysaccharide (LPS), and β-glucan are all recognized as established inducers of trained immunity, and they are used in clinical applications. BCG not only effectively prevents tuberculosis but also enhances bone marrow hematopoietic progenitors, boosts the antimicrobial activity of neutrophils, and stimulates emergency granulopoiesis through G-CSF signaling. This process helps protect newborn animals from sepsis-related challenges [100,102,103]. Evidence from human trials by Cirovic et al. highlights that BCG vaccination induces central trained immunity by reprogramming bone marrow HSPCs, characterized by the transcriptional activation of neutrophil-lineage genes (e.g., S100A family and SERPINA1). Correspondingly, clinical observations show a marked elevation of circulating neutrophil levels in neonates following BCG administration. This systemic immune rewiring ultimately bolsters broad-spectrum defense against non-specific infectious challenges [104]. Beyond BCG, other trained immunity inducers have also demonstrated considerable translational potential. Research revealed that low-dose LPS preconditioning increases a specific neutrophil subset (e.g., the N3 subset) distinguished by elevated phagocytic and bactericidal capabilities, upregulates galectin-3 expression, and markedly improves host resistance to fatal Pseudomonas aeruginosa infection. This method has been verified in patients with acute respiratory failure, demonstrating that galectin-3 levels positively correlate with neutrophil activity and patient survival, highlighting the translational potential of trained immunity in the clinical prevention and treatment of sepsis [105]. Despite the widespread application of fungal-derived β-glucan as a trainer of the innate immune system, conclusive human-based evidence specifically regarding its impact on neutrophils is currently lacking, as most existing data are extrapolated from murine models. While experimental evidence, such as the cross-protection observed with heat-inactivated Cryptococcus mutants across different mouse models, underscores the potential contribution of innate effectors like neutrophils, the translation of these results into human physiological settings remains a critical area for future investigation [106]. Furthermore, individual responses to trained immunity inducers exhibit significant heterogeneity. For instance, data from BCG-vaccinated cohorts indicate that approximately 24% of individuals fail to elicit a typical trained immunity response [107]. This heterogeneity further suggests that the underlying mechanisms governing the induction and maintenance of trained immunity are not universally conserved, and their biological foundations and reproducibility warrant more rigorous investigation. These challenges, to some extent, constrain the clinical translation of trained immunity and underscore the urgent need to develop personalized intervention strategies.

### 4.2. Regulation of Macrophages by Trained Immunity in Infectious Diseases

**Figure 4:**
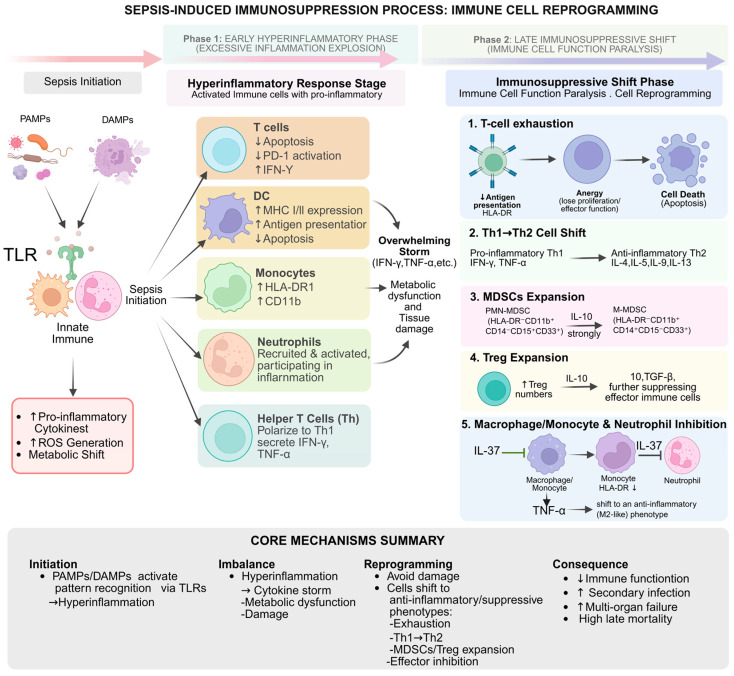
Immunosuppression mediated by sepsis: a biphasic model of immune cell reprogramming. Sepsis commences with the detection of Pathogen-Associated Molecular Patterns (PAMPs) and Damage-Associated Molecular Patterns (DAMPs) by Toll-like Receptors (TLRs), instigating the initial hyperinflammatory phase. During this phase, T cells, dendritic cells (DCs), monocytes, neutrophils, and T helper 1 (Th1) cells are vigorously stimulated, resulting in a cytokine storm that causes metabolic dysfunction and tissue damage. Subsequently, the immune system enters the late immunosuppressive phase, marked by significant immune cell reprogramming events, including T-cell exhaustion and anergy, a Th1-to-Th2 phenotypic shift, proliferation of myeloid-derived suppressor cells (MDSCs) and regulatory T (Treg) cells, along with functional inhibition of macrophages, monocytes, and neutrophils. This adaptive reprogramming ultimately leads to immunological paralysis, heightening the vulnerability of sepsis patients to subsequent infections, multiple organ failure, and late-stage mortality. Figure created using Biorender.

Macrophages, as terminally developed cells of the monocyte lineage, have significant flexibility and dynamically adjust their functional status in response to microenvironmental signals, typically classified into pro-inflammatory (M1) and anti-inflammatory, pro-repair (M2) phenotypes [33]. During the initial phases of sepsis, macrophages often polarize to the M1 phenotype, eradicating pathogens by the secretion of pro-inflammatory cytokines, including tumor necrosis factor-alpha (TNF-α), interleukin-1β (IL-1β), and IL-6 [108,109]. As the disease advances to later stages, persistent inflammatory stimulation encourages a transition to the M2 phenotype to facilitate tissue healing; nevertheless, excessive M2 polarization may result in immunological paralysis [110]. In the advanced stages of sepsis, macrophages display epigenetic modifications, such as changes in H3K4me3 and H3K9me3, which inhibit the production of host defense genes, resulting in cellular dysfunction [5]. The deviations in polarization, along with epigenetic remodeling, form the fundamental mechanisms that lead to immunological paralysis and heightened vulnerability to secondary infections in late-stage sepsis, a state often linked to significantly elevated mortality rates [111] (Figure 4).

Trained immunity enhances immunological responses through the modulation of macrophage metabolic reprogramming and phenotypic polarization, presenting a new intervention strategy to counteract sepsis-related immunosuppression and improve host antimicrobial defenses [3,112]. Now, numerous inducers of trained immunity have been recognized, encompassing traditional inducers like microbial-associated molecular patterns (e.g., fungal β-glucan) and Bacillus Calmette-Guérin (BCG) [2,112,113], alongside novel inducers such as Oroxylin A, BCG-derived outer membrane vesicles (B-OMVs), and inactivated pathogens [114,115] roxylin A stimulates trained immunity in macrophages by affecting the Dectin-1-Syk axis, facilitating LC3-associated phagocytosis (LAP) alongside increased glycolytic activity and mTOR pathway activation, therefore augmenting macrophage phagocytic and bactericidal efficacy. Macrophages conditioned with Oroxylin A provide protective benefits via adoptive transfer, demonstrating substantial protective effects in both lipopolysaccharide (LPS)-induced and cecal ligation and puncture (CLP) sepsis murine models [114]. B-OMVs activate the AKT-mTOR-HIF-1α signaling pathway through a TLR2-dependent mechanism, resulting in metabolic reprogramming in macrophages characterized by increased aerobic glycolysis, while concurrently facilitating hematopoietic stem cell proliferation and myelopoiesis to elicit central trained immunity. Their strong protective efficacy against polymicrobial sepsis has been evidenced in CLP models, endorsing B-OMVs as a viable immunotherapeutic approach with prospective uses in sepsis vaccination and the treatment of other infectious diseases [115].

It has been established through human challenge studies that BCG-induced trained immunity provides cross-protection against viral infections. This process is mediated by the epigenetic landscape modification of monocytes, specifically H3K27ac. The resultant suppression of viremia correlates with a potentiation of IL-1β responses rather than specific T-cell immunity. Furthermore, genetic and functional evidence identify IL-1β as a master regulator of this memory, influencing H3K4me3/H3K9me3 marks. This paradigm establishes a robust link between innate immune memory and clinical resistance to pathogens [116].

The advantages of trained immunity are not limited to circulating monocytes; they also encompass tissue-resident macrophages, with hematopoietic stem cells playing a crucial role in mediating systemic and enduring effects through central trained immunity [2]. Trained immunity within diverse tissue-resident macrophage populations exhibits considerable tissue specificity and functional diversity. For instance, pulmonary tissue-resident macrophages can independently develop local trained immunity memory, independent of bone marrow stem cells. Nevertheless, their protective effects are pathogen-specific, offering effective defense against Streptococcus pneumoniae but not against SARS-CoV-2 [117]. Conversely, following recovery from SARS-CoV-2 infection, alveolar macrophages undergo persistent epigenetic remodeling, characterized by increased chromatin accessibility at genes linked to the type I interferon cascade, thus establishing antiviral trained immunity. Following heterologous influenza A virus infection, these cells significantly enhance interferon-stimulated gene expression, substantially reducing the severity of secondary infections and lowering mortality, a process that is preserved in humans [57]. Furthermore, synovial macrophages can develop trained immunity after LPS pre-stimulation, as evidenced by increased histone H3 trimethylation levels and metabolic reprogramming mediated by the mTOR-HIF1α pathway. Upon Staphylococcus aureus infection, this conditioned memory induces CX3CR1^+^ macrophage-mediated hyperinflammation and osseous tissue destruction, pathological injuries that can be significantly alleviated by the mTOR inhibitor rapamy [118].

In essence, neutrophils and macrophages, the principal effector cells of the innate immune system, undergo functional remodeling through trained immunity in response to infectious diseases, thereby constituting a crucial defense against infection. Neutrophils, leveraging their numerical dominance and rapid response capabilities, can significantly enhance their bactericidal function and disease tolerance via the induction of trained immunity; macrophages, owing to their considerable phenotypic plasticity and tissue-resident attributes, regulate immune homeostasis under the influence of trained immunity, mediating a balance between pathogen elimination and tissue repair processes. The role of trained immunity in infectious diseases reveals a unique “double-edged sword” effect: moderate activation can facilitate pathogen clearance and improve disease outcomes, whereas excessive or inappropriate activation may lead to maladaptive reprogramming and pathological damage. Therefore, future investigations should focus on elucidating specific intervention strategies for educational immunity to enhance antimicrobial functions while effectively preventing pathological inflammation and immune paralysis in severe infections such as sepsis. Simultaneously, by employing advanced technologies like single-cell sequencing and metabolomics, we aim to clarify the molecular mechanisms through which trained immunity regulates these two cell types and to delineate their synergistic regulatory patterns during infection, thus providing novel theoretical foundations and technical approaches for the precise prevention and treatment of infectious diseases.

## 5. Hypoxia and Trained Immunity: Neutrophils and Macrophages

### 5.1. Hypoxia-Induced Maladaptive Reprogramming of Neutrophil Trained Immunity

**Figure 5:**
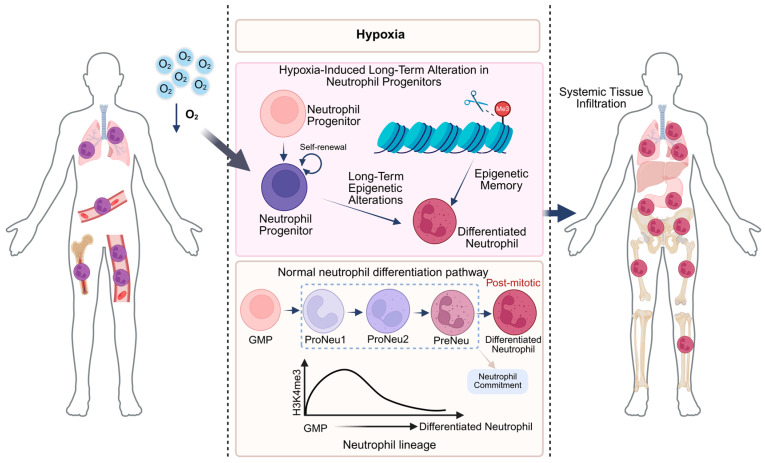
Hypoxia induces sustained maladaptive immune reprogramming of neutrophils via histone H3 clipping and H3K4me3 loss in neutrophil progenitors. In the bone marrow, self-renewing neutrophil-committed progenitors (GMP → ProNeu1 → ProNeu2 → PreNeu) are directly affected by systemic hypoxia (e.g., caused by ARDS or high-altitude exposure). The lysine residue necessary for H3K4 trimethylation is eliminated when hypoxia causes the N-terminal domain of histone H3 (H3) to be clipped. This causes the activating mark to be widely lost. H3K4me3 levels in mature neutrophils are eventually sustainedly reduced as a result of this epigenetic modification, which is spread by mitosis to downstream differentiation stages. As a result, their tissue infiltration capacity is changed, their effector functions are permanently compromised, and they become more vulnerable to subsequent infections. Adapted with permission from [121], copyright 2025 Springer Nature.

Research utilizing a mouse model of hypoxic acute lung injury has shown that systemic hypoxia alone can acutely influence neutrophil effector functions by altering bone marrow myelopoiesis, resulting in hyperinflammatory neutrophils in peripheral tissues and consequently worsening tissue damage [119]. Recent findings indicate that systemic hypoxia caused by acute respiratory distress syndrome (ARDS) leads to prolonged dysfunction of circulating neutrophil effector capabilities in patients during the 3- to 6-month recovery phase. This is marked by reduced antimicrobial efficacy, including decreased expression and secretion of granule proteins (e.g., alpha-1-antitrypsin) and compromised opsonic phagocytosis, ultimately heightening vulnerability to secondary bacterial infections. As previously mentioned, in BCG-induced trained immunity, the antimicrobial function of neutrophils is governed by the levels of H3K4me3 [120]. Chromatin immunoprecipitation sequencing (ChIP-seq) analysis of the circulating neutrophil population in ARDS survivors 3 to 6 months post-hospitalization demonstrated markedly diminished H3K4me3 levels at genes linked to neutrophil effector functions (such as granule protein maturation, calcium signaling, and inflammatory responses), suggesting hypoxia as a pivotal regulator of epigenetic reprogramming in human neutrophils. Consistent results were derived from neutrophils of patients subjected to high-altitude hypoxemia. In mouse models of systemic hypoxia and pulmonary damage, the decrease in H3K4me3 levels arises from neutrophil-committed progenitors (proNeu, preNeu), which experience histone H3 N-terminal clipping under hypoxic conditions, leading to the loss of the trimethylation site [121]. These findings collectively demonstrate that systemic hypoxia induces enduring maladaptive reprogramming of neutrophil immunity by initiating histone H3 clipping and the subsequent loss of H3K4me3 in neutrophil progenitor cells (Figure 5).

This process is quite different from how neutrophils are reprogrammed by substances like BCG or β-glucan. These substances increase H3K4me3 levels, which then improves their ability to fight infections. In a rat model of low oxygen levels, BCG immunization partially restored H3K4me3 levels and improved the neutrophils’ ability to fight infections. These findings suggest that different environmental factors, such as low oxygen, infection, and metabolic signals, can change the epigenetic profile of bone marrow progenitors. This change can either help or hinder the development of neutrophils, which affects the balance between the body’s ability to fight infections and its vulnerability to disease. In neutrophils, the hypoxia-inducible factor (HIF) is the main regulator of how cells adapt to low oxygen. It controls tissue survival, important functions, and key metabolic processes [119,122,123]. The epigenetic landscape is both a target and a regulator of HIF activity, implying that the interplay between HIF and trained immunity signaling pathways may be critical for the reciprocal regulation of trained immunity. Therefore, employing trained immunity inducers such as BCG and β-glucan to reinstate H3K4me3 levels in neutrophils could offer a potentially beneficial therapeutic strategy to alleviate immune dysfunction associated with hypoxia-related ailments—including ARDS, chronic obstructive pulmonary disease (COPD), and severe COVID-19—by reversing detrimental epigenetic reprogramming.

### 5.2. Dual Functions of Hypoxia in Regulating Macrophage Trained Immunity

The regulation mechanism of hypoxia on macrophage-trained immunity closely resembles that seen in neutrophils, principally altering macrophage response to secondary stimuli via metabolic reprogramming and epigenetic remodeling. As circulatory precursors, monocytes exhibit metabolic continuity during their differentiation into tissue macrophages; however, significant differences exist between the two regarding the stability of their trained immunity phenotypes and their dependency on the microenvironment. Macrophages, in particular, rely more heavily on tissue-specific signals to maintain their functional states. During this process, hypoxia-inducible factor 1-alpha (HIF-1α) functions as a pivotal hub integrating hypoxic and metabolic signals [124]. While hypoxia stabilizes its expression, Cheng et al. demonstrated through ex vivo experiments on human monocytes that trained stimuli such as β-glucan can also activate HIF-1α via the Akt/mTOR pathway. This activation drives metabolic reprogramming characterized by aerobic glycolysis (also known as Warburg-like glycolysis), the pentose phosphate pathway, and glutaminolysis. Subsequently, Arts et al. further established that this metabolic shift not only fulfills cellular energy requirements but also modulates the activity of epigenetic enzymes by generating key metabolic intermediates such as fumarate. The accumulation of fumarate inhibits the KDM5 family of demethylases and facilitates the maintenance of activating histone modifications, specifically H3K4me3 [125]. This mechanism ensures that chromatin loci associated with anti-infection, pro-inflammatory, and tissue repair genes remain in an open state, ultimately establishing a trained immunity phenotype characterized by epigenetic memory. Infection with severe acute respiratory syndrome coronavirus 2 (SARS-CoV-2) prompts a metabolic transition from oxidative phosphorylation to glycolysis, resulting in succinate buildup. This metabolic remodeling simulates the hypoxic microenvironment and stabilizes HIF-1α, accompanied by epigenetic alterations such as histone acetylation and succinylation. These modifications direct macrophages towards a chronically hyperactivated trained immunity phenotype, enhancing inflammatory responses and facilitating the pathogenesis of cytokine storms in COVID-19 [126].

In hypoxic settings, HIF-1α overexpression directly influences immune-related gene expression and alters chromatin architecture by changing the activity of histone acetylation and methylation enzymes [127]. Recent investigations have discovered a new metabolic oxygen-sensing route in macrophages—the PNPO-PLP axis—that functions independently of traditional HIF signaling. This pathway detects prolonged hypoxia and facilitates metabolic reprogramming by modulating lysosomal function and polysulfide production, thereby promoting a pro-inflammatory phenotype. These findings provide a novel avenue for clarifying the metabolic memory mechanisms that underpin trained immunity in chronic hypoxia [128]. The control of macrophages by hypoxia demonstrates a dual nature: acute or physiological hypoxia can elicit protective trained immunity, while chronic or pathological hypoxia typically induces immunological tolerance or functional dysregulation [129]. Hypoxia-preconditioned stromal progenitors secrete insulin-like growth factor 2 (IGF-2), which directs macrophages to adopt a persistent anti-inflammatory phenotype via metabolic reprogramming reliant on mitochondrial oxidative phosphorylation, thus providing tissue protection in the hypoxic microenvironment. In contrast, persistent hypoxia can stimulate macrophages to produce decreased nicotinamide adenine dinucleotide phosphate (NADPH) through the non-oxidative pentose phosphate pathway, hence improving the efferocytosis of apoptotic neutrophils. The “prime-and-ready” bimodal paradigm posits that trained immunity in hypoxic microenvironments facilitates the effective clearance of apoptotic cells while mitigating oxidative stress damage via pre-transcriptional priming of phagocytosis-related genes and a reservoir of reducing capacity, providing a novel theoretical framework for tissue homeostasis maintenance [130].

In summary, hypoxia exerts a complex bidirectional influence on the trained immunity of neutrophils and macrophages. Pathological hypoxia frequently results in maladaptive trained immunity, characterized by diminished neutrophil antimicrobial efficacy or heightened macrophage activation, hence intensifying infection and inflammatory responses in hypoxia-associated conditions such as ARDS and COVID-19. In contrast, physiological or preconditioning hypoxia can elicit protective trained immunity, such as through IGF-2-mediated anti-inflammatory macrophage polarization or improved efferocytosis of apoptotic cells. Future research should investigate the precise mechanisms through which hypoxia-inducible factors (HIFs) and emerging oxygen-sensing pathways, such as the PNPO-PLP axis, govern epigenetic reprogramming and metabolic remodeling, especially their effects on histone modifications like H3K4me3 and subsequent immune gene expression. By specifically targeting these molecular nodes, we can devise innovative therapeutic strategies, such as employing trained immunity inducers like BCG to restore H3K4me3 levels and antimicrobial functionality in neutrophils from patients with hypoxia-related conditions, or achieving precise inflammatory rebalancing through intervention in macrophage metabolic and epigenetic states. This would not only alleviate the immune paralysis linked to hypoxia-related disorders such as ARDS, chronic obstructive pulmonary disease (COPD), and severe COVID-19, but also offer new perspectives for comprehending and addressing other chronic inflammatory diseases.

## 6. Ischemia-Induced Trained Immunity: Neutrophils and Macrophages

### 6.1. Ischemia-Induced Trained Immunity in Neutrophils

Neutrophils, the body’s first line of defense against ischemic injury, have many functions and complex behaviors. When ischemia occurs, these cells quickly move to the affected areas. There, they help the healing process by releasing neutrophil extracellular traps (NETs), which remove damaged tissue, and by engulfing and digesting cellular debris. Excessive or prolonged NET release in ischemic conditions, such as stroke and myocardial infarction, can exacerbate thrombosis and immune suppression, heightening the risk of secondary infections [131], while also impeding angiogenesis and vascular remodeling [132]—processes closely linked to poor prognosis in ischemic diseases.

A study examining cardiac ischemia and bone marrow neutrophil production reveals that ischemia significantly enhances myeloid-biased differentiation towards the neutrophil lineage in the bone marrow, indicating a prolonged impact of ischemia on neutrophils. Transcriptomic analysis indicates that ischemia enhances signaling pathways related to phagocytosis, degranulation, and cytokine signaling in bone marrow neutrophils, implying that ischemia functionally reprograms neutrophils to display an improved functional phenotype, potentially reliant on the cGAS-STING pathway [133]. The findings suggest that ischemic conditions are a significant environmental element that might induce trained immunity, reprogramming neutrophils via pathways including hypoxia-inducible factor (HIF-1α) to exhibit increased reactivity to subsequent stimuli.

In a cardiac ischemia–reperfusion paradigm, a single-cell transcriptome study has shown a cardioprotective subpopulation of neutrophils—Ym-1hi neutrophils [134]. This subset demonstrates significant growth during the late reperfusion phase, transitioning functionally from a classical pro-inflammatory phenotype to a reparative phenotype, marked by elevated expression of signature molecules like Ym-1 and the ability to promote macrophage polarization towards the reparative M2 phenotype, thereby enhancing cardiac function and decreasing infarct size. Crucially, experimental evidence demonstrates that the cardioprotective impact is directly correlated with the relative abundance of Ym-1hi neutrophils, rather than with absolute neutrophil levels. This discovery indicates that ischemia may prompt neutrophils to develop an immune memory phenotype, enabling them to facilitate long-term tissue repair via trained immunity mechanisms. This presents a new research perspective and theoretical basis for therapeutic strategies aimed at specific neutrophil subsets in ischemic heart disease.

### 6.2. Ischemia-Induced Trained Immunity in Macrophages

Ischemia, or the release of damage-associated molecular patterns (DAMPs) due to ischemia, can trigger trained immune responses in monocytes and macrophages. A study on how monocytes change function after ischemic conditioning showed that a short ischemic event, like a 24 h femoral artery blockage, can prepare monocytes from the bone marrow. This preparation improves their ability to help restore blood flow and create new blood vessels during later, lasting ischemia [135]. This process is closely linked to metabolic changes and histone modifications in monocytes, particularly the significant increase in hypoxia-inducible factor-1α (HIF-1α) and its target gene, glucose transporter 1 (GLUT-1). These findings offer new possibilities for developing cell-based treatments for ischemic vascular diseases.

Ischemia-Reperfusion Injury (IRI) is a damaging process that occurs when blood flow returns to tissues that were previously deprived of it. This renewed blood flow worsens inflammation and increases tissue damage. During ischemia-reperfusion injury (IRI), macrophages, which are crucial to the disease process, show changes in their characteristics and functions during both the injury and recovery phases. Research indicates that giving antibiotics beforehand (ABX) significantly reduces the effects of hepatic ischemia-reperfusion injury (IRI) by changing the gut microbiota. The protective effect was closely associated with increased levels of alpha-ketoglutarate (α-KG), a metabolite produced by the gut microbiota. This metabolite promoted the shift in macrophages from a pro-inflammatory M1 state to a reparative M2 state through metabolic changes. As a result, liver function improved, inflammatory responses decreased, and apoptosis was reduced. These findings suggest that targeting macrophage metabolism could be a useful treatment for IRI [136]. When muscle tissue is damaged by a lack of blood flow, endothelial cells, which are known to have increased glycolytic activity, produce and release lactate. Macrophages then take up this lactate through monocarboxylate transporter 1 (MCT1), which helps them reprogram metabolically and functionally, moving them toward an M2-like reparative state. Polarized macrophages contribute to sustained regeneration and vascular repair in ischemic muscle by fostering the proliferation of myogenic precursor cells and augmenting the release of vascular endothelial growth factor (VEGF) [137]. During the post-myocardial infarction repair phase, TREM2^+^ macrophages undergo metabolic reprogramming following the efferocytosis of apoptotic cardiomyocytes, which promotes the storage and release of itaconate. This immunomodulatory metabolite, itaconate, mitigates cardiomyocyte apoptosis in areas of ischemic damage and stimulates cardiac fibroblast proliferation, thereby improving cardiac morphology and function. Studies show that the trained immunity of macrophages is crucial for tissue repair and the growth of new blood vessels in conditions caused by insufficient blood flow [138].

**Figure 6:**
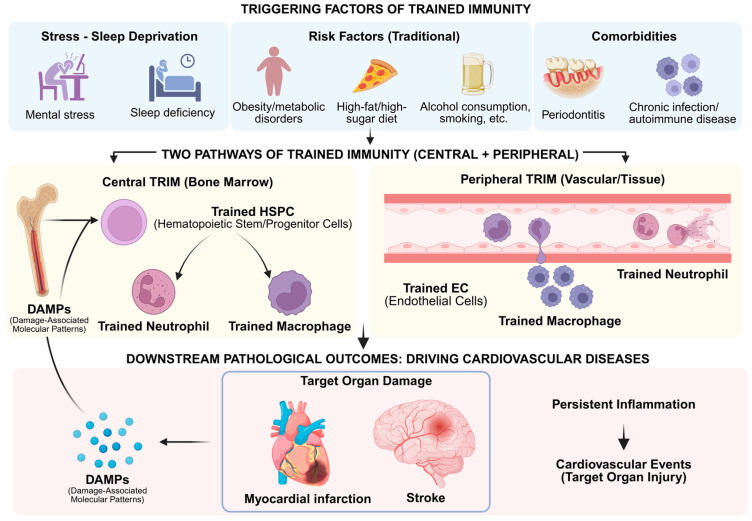
Trained immunity drives Atherosclerotic Cardiovascular Disease. Cardiovascular risk factors, unhealthy lifestyles, and comorbidities cause TRIM, which manifests peripherally in innate immune/endothelial cells and centrally in bone marrow HSPCs. Atherosclerosis is encouraged by trained cells, and a vicious cycle is created when plaque rupture causes acute events and the production of DAMPs, which intensify central TRIM. Figure created using Biorender.com.

Maladaptive trained immunity in macrophages plays a critical role in the progression of chronic inflammatory disorders, particularly atherosclerotic cardiovascular disease (ASCVD). Various risk factors, including hyperlipidemia, hyperglycemia, and endogenous atherogenic stimuli like oxLDL or endotoxins, can trigger training in bone marrow hematopoietic stem/progenitor cells (HSPCs) [139] (Figure 6). This central reprogramming facilitates the release of trained monocytes that, upon migrating to the arterial wall and differentiating into macrophages, exhibit an exacerbated inflammatory response to local plaque stimuli (e.g., oxLDL), thereby accelerating plaque progression and destabilization [46].

Human studies have further corroborated this paradigm, showing that circulating monocytes in patients with coronary atherosclerosis are characterized by a pre-trained state. These cells demonstrate enhanced pro-inflammatory responses upon ex vivo stimulation with LPS or oxLDL, coupled with elevated H3K4me3 and H3K27ac occupancy at pro-inflammatory gene promoters. Furthermore, acute ischemic events, such as myocardial infarction (MI), can exacerbate atherosclerotic progression by inducing systemic epigenetic rewiring in HSPCs and monocytes—for instance, via KMT5a-mediated H4K20me3 modification that activates the Syk pathway. The clinical efficacy observed in major trials (e.g., CANTOS and COLCOT), which demonstrated that targeting IL-1β or downstream inflammatory pathways significantly reduces the recurrence of cardiovascular events, provides robust clinical validation for the pathological role of trained immunity in atherosclerosis [140]. Collectively, these findings establish the potential of reversing maladaptive innate memory as a novel therapeutic strategy for cardiovascular disease.

**Figure 7:**
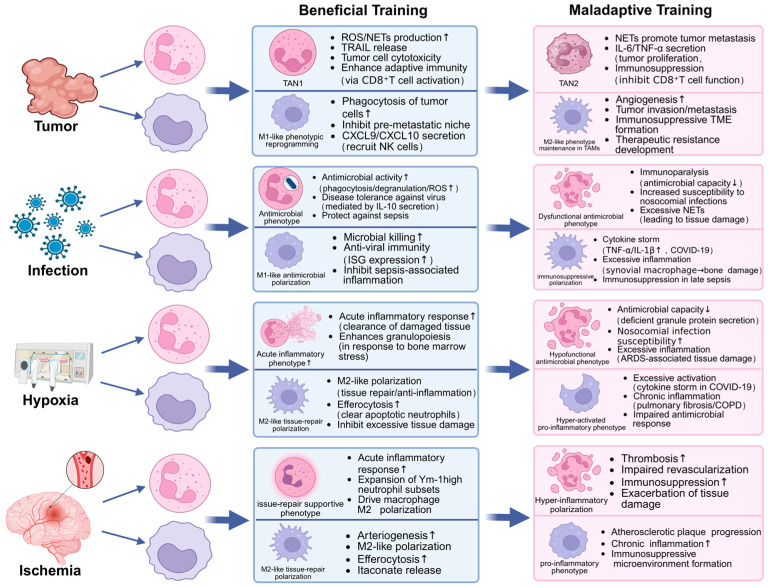
Schematic depiction of the regulatory mechanisms and pathological ramifications of trained immunity in neutrophils and monocytes/macrophages. This image illustrates the reciprocal regulatory effects of pathogenic stimuli and the dual pathophysiological functions of trained immunity in neutrophils and monocytes/macrophages, the primary effector cells of the innate immune system. Trained immunity in these cell populations is primarily regulated by epigenetic reprogramming, marked by the accumulation of activating histone modifications such as H3K4me3 and H3K27ac, alongside metabolic remodeling, particularly a transition from oxidative phosphorylation to aerobic glycolysis driven by the mTOR-HIF-1α pathway. In neutrophils, trained immunity primarily occurs within bone marrow hematopoietic stem and progenitor cells, while monocytes/macrophages demonstrate both central (bone marrow-derived) and peripheral (tissue-specific) reprogramming, with tissue-resident subsets exhibiting distinct functional phenotypes. Hypoxia and ischemia have reciprocal regulatory influences on trained immunity in these cells: pathogenic stimuli may trigger maladaptive reprogramming, whilst traditional inducers of trained immunity, such as BCG and β-glucan, augment their protective immunological functions. Trained immunity serves as a double-edged sword: it can enhance anti-infective and anti-tumor responses while facilitating tissue repair after ischemic injury; nevertheless, inappropriate reprogramming may lead to persistent inflammation, immunological paralysis, and tumor growth. Targeting the epigenetic and metabolic checkpoints of trained immunity represents a viable technique for the precise control and therapy of associated clinical diseases. In the figure, ↑ denotes an increase or upregulation, and ↓ denotes a decrease or downregulation of the listed biological parameters. Figure created using Biorender.com.

In summary, ischemia, a complex pathophysiological process, displays as acute cellular injury and acts as a “training stimulus” that prepares neutrophils and macrophages for long-term functional reprogramming. This ischemia-induced trained immunity functions as a double-edged sword, with its overall impact contingent upon the exact cell subset engaged, alongside the severity and duration of the initiating stimulation. Future research should focus on elucidating the precise regulatory pathways that control trained immunity, analyzing the functional diversity and intercellular interactions among various cell subsets, and investigating targeted intervention strategies that leverage its advantageous effects while reducing harmful consequences. These efforts will establish a more substantial theoretical basis and clinical justification for precision treatments in ischemic disorders (Figure 7).

## 7. Conclusions and Perspectives

The idea that the innate immune system does not have immunological memory has been significantly changed by the concept of “trained immunity”. Innate immune memory is characterized by the sustained functional reprogramming of innate immune cells, including neutrophils and monocytes/macrophages, following encounters with pathogens, vaccines, or endogenous stimuli. This phenomenon is mediated by enduring epigenetic remodeling and metabolic reconfiguration. This review has thoroughly analyzed the pivotal functions of neutrophils and monocytes/macrophages in trained immunity, the molecular mechanisms involved, and their dual protective and pathological roles in various pathophysiological scenarios, including infection, cancer, and hypoxic–ischemic conditions.

Trained immunity fundamentally operates through a substantial interplay of epigenetic changes and metabolic reprogramming. The enduring presence of favorable histone modifications, such as H3K4me3 and H3K27ac, at the promoters of pro-inflammatory genes, constitutes the epigenetic basis for the long-lasting memory observed. Simultaneously, a transition to aerobic glycolysis, influenced by the mTOR-HIF-1α pathway, fulfills the energetic and biosynthetic requirements of activated cells while producing metabolites—such as succinate, itaconate, and lactate—that directly regulate the function of histone-modifying enzymes, thus connecting metabolic status with the epigenetic landscape. This reprogramming extends beyond mature effector cells and can be instilled at the level of bone marrow progenitors, creating a lasting, multifaceted functional remodeling that is essential for comprehending how transient cells such as neutrophils can display memory.

Neutrophils, once considered transient and incapable of memory, demonstrate considerable functional diversity and plasticity upon the induction of trained immunity. Consequently, this leads to an enhanced capacity for reactive oxygen species production, phagocytosis, NETosis, and cytokine release upon re-exposure, as well as a polarization towards an anti-tumor N1 phenotype within the tumor microenvironment. For example, β-glucan-induced trained granulopoiesis, facilitated by type I interferon signaling, might provide transferable anticancer benefits through bone marrow transplantation, highlighting the persistence of central trained immunity. This neutrophil memory presents a dual challenge. In severe conditions such as sepsis and ARDS, maladaptive training may cause a reduction in H3K4me3 at essential antimicrobial gene promoters, leading to functional depletion and increased vulnerability to secondary infections. Hypoxia-induced cleavage of histone H3 has been recognized as a mechanism that drives pathological reprogramming, underscoring how microenvironmental signals can adversely influence the trajectory and strength of trained immunity.

Monocytes and macrophages, owing to their inherent tissue residency and phenotypic plasticity, serve as prime models for the study of trained immunity. This training process can induce polarization of these cells towards an M1-like phenotype, thereby enhancing antigen presentation, phagocytic function, and chemokine release, which in turn offers protection during anti-tumor and anti-infective responses. Recent research substantiates that trained immunity can induce localized memory in tissue-resident macrophages. Alveolar macrophages from SARS-CoV-2-infected mice demonstrate antiviral training, marked by enhanced chromatin accessibility at transcription factor binding sites for the IRF family, providing enduring protection against heterologous infections. In contrast, abnormal induction of trained immunity is a primary factor in chronic inflammatory disorders. Oxidized low-density lipoprotein (oxLDL) and cholesterol crystals, both produced by the body, can worsen inflammation in monocytes and macrophages. This increased inflammation can then worsen atherosclerosis, obesity, and diabetes.

The trained immunity of neutrophils and monocytes/macrophages is critically interdependent, requiring combined regulation. Common inducers, such as β-glucan and BCG, can activate shared pathways in both types of cells. Co-programming at the level of bone marrow progenitors, coupled with ensuing intercellular signaling, exerts an influence on the tumor microenvironment and modulates responses to infection. The result of this complex interaction is determined by several factors, including the availability of oxygen, the body’s metabolic state, and epigenetic changes. These factors together decide whether the outcome is beneficial or harmful.

Despite these advancements, substantial gaps in understanding remain. The cellular heterogeneity of trained immunity remains inadequately investigated; specific fractions of neutrophils and monocytes/macrophages presumably exhibit various training attributes and regulation mechanisms. Current research has mostly focused on populations, which means we need to understand epigenetic and metabolic processes at the single-cell level. Because trained immunity has both innate and adaptive features, we need to develop specific treatment strategies for clinical use. A significant issue is to improve host defense during infections without provoking hyperinflammation, or to selectively promote anti-tumor phenotypes while circumventing pro-tumorigenic consequences. Thirdly, further investigation is warranted into the synergistic interplay between trained and adaptive immunity. While it is understood that trained myeloid cells can modulate T cell and NK cell responses through cytokine secretion and antigen presentation, the specific signaling pathways and interaction networks governing this crosstalk remain incompletely defined.

Future research endeavors ought to employ advanced methodologies, such as single-cell sequencing, metabolomics, and epigenomics, to enable detailed examinations of the molecular regulatory networks that orchestrate trained immunity within neutrophils and monocytes/macrophages. A primary objective is to elucidate the context-specific phenotypic attributes and regulatory targets linked to trained immunity across a spectrum of disease states. Simultaneously, efforts to translate research must be amplified to create highly targeted, low-toxicity modulators of trained immunity. The exploration of rational combination strategies, including the use of “trained immunity inducers in conjunction with immune checkpoint inhibitors” in oncology and “trained immunity modulators alongside standard-of-care” in the context of infectious and inflammatory diseases, presents an opportunity to develop a new theoretical framework and therapeutic options. These approaches could facilitate the precise prevention and treatment of a range of human diseases.

## Figures and Tables

**Table 1:** Key findings, limitations, and conflicting results in human clinical studies of trained monocytes and macrophages.

Study Domain	Key Findings	Major Limitations	Conflicting Results and Controversies
Vaccination (e.g., BCG, Oral Polio)	Induction of epigenetic (H3K4me3) and metabolic (glycolysis) reprogramming; provides non-specific protection against heterologous infections.	Short follow-up durations (mostly <1 year); reliance on peripheral blood as a surrogate for central immunity.	Discrepancies in the efficacy of BCG for preventing COVID-19 infections across different large-scale trials (e.g., ACTIVATE trial vs. others).
Metabolic Stimuli (Western diet, oxLDL)	Chronic exposure to endogenous or dietary stimuli leads to persistent pro-inflammatory monocyte phenotypes; linked to atherosclerosis risk.	Small sample sizes in clinical cohorts; difficulty in isolating the specific contribution of training from baseline genetic factors.	Uncertainty regarding causality: whether trained immunity is a primary driver or a secondary bystander in chronic metabolic diseases.
Clinical Interventions and Sepsis	Identification of post-sepsis immunoparalysis characterized by epigenetic repression and reduced cytokine production.	Ethical constraints in repeated bone marrow sampling; high inter-patient heterogeneity in inflammatory responses.	Variable persistence of the trained phenotype in vivo vs. ex vivo; inconsistent responses to different TLR ligands during restimulation.
Methodology and Populations	Age and sex significantly influence the magnitude and direction of the trained immune response.	Lack of standardized biomarkers; interference from prior microbial and environmental exposures on current immune status.	Controversies regarding the long-term stability of the trained phenotype and its rate of decline after the initial stimulus is removed.

Data synthesized from references [45,46,47,48,49,50].

## Data Availability

No new data were created or analyzed in this study.
